# Interface between falls and perceptions of quality of life of older adults: a qualitative study

**DOI:** 10.1590/0034-7167-2025-0007

**Published:** 2025-12-08

**Authors:** Caio Leonardo Faria Andrade, Patrícia Chaves da Silva, Kevin Silva Moreira, Jonathan Ballico de Moraes, Juliana Balbinot Reis Girondi, Newton Ferreira de Paula

**Affiliations:** I Fundação Presidente Antônio Carlos. Uberlândia, Minas Gerais, Brazil; II Universidade Estadual de Goiás. Itumbiara, Goiás, Brazil; III Universidade Federal de Santa Catarina. Florianópolis, Santa Catarina, Brazil

**Keywords:** Accidental Falls, Quality of Life, Aged, Frailty, Vulnerability., Accidentes por Caídas, Calidad de Vida, Anciano, Fragilidad, Vulnerabilidad.

## Abstract

**Objectives:**

to understand the relationship between falls and perceptions of quality of life among older adults.

**Methods:**

this is a descriptive and exploratory study with a qualitative approach, conducted with older adults in the Family Health Strategy of Itumbiara, Goiás, using Bardin’s content analysis.

**Results:**

analysis of interviews with 26 older adults revealed four thematic axes: Fall dynamics, circumstances and associated factors; Physical and emotional repercussions of falls; Spirituality and social support network; and Perceptions and suggestions for a safer life. Falls increase older adults’ fragility and vulnerability, affect their health, dependence and functional capacity and tend to be normalized and trivialized by older adults themselves.

**Final Considerations:**

falls impact older adults’ physical and emotional health. It is necessary to raise awareness about the causes, consequences and prevention of this event, in order to promote healthy and active aging.

## RESUMEN

**Objetivos:**

comprender la relación entre las caídas y la percepción de la calidad de vida en adultos mayores.

**Métodos:**

estudio descriptivo y exploratorio con enfoque cualitativo, realizado con adultos mayores de la Estrategia de Salud Familiar de Itumbiara, Goiás, mediante el Análisis de Contenido de Bardin.

**Resultados:**

el análisis de entrevistas con 26 adultos mayores reveló cuatro ejes temáticos: Dinámica de las caídas, circunstancias y factores asociados; Repercusiones físicas y emocionales de las caídas; Espiritualidad y red de apoyo social; y Percepciones y sugerencias para una vida más segura. Las caídas aumentan la fragilidad y vulnerabilidad de las personas mayores, afectan su salud, dependencia y capacidad funcional, y tienden a ser normalizadas y trivializadas por las propias personas mayores.

**Consideraciones Finales:**

las caídas impactan la salud física y emocional de las personas mayores. Es necesario concienciar sobre las causas, consecuencias y prevención de este evento para promover un envejecimiento saludable y activo.

## INTRODUCTION

Older adults are in a continuous process of biopsychosocial and spiritual development, enriched by considerable life experience. Although physiological changes typical of aging do not necessarily constitute diseases, they can increase fragility and vulnerability to certain illnesses. On the other hand, many older adults reach old age with chronic noncommunicable diseases acquired throughout adulthood, which increases fragility and the risk of experiencing, for instance, falls^(1)^.

A fall can be understood as unintentional contact with the ground or other supporting surface, resulting from a change in individuals’ position to a lower level than that at the time prior to this event^(2)^. It can result in reduced functional capacity, compromised autonomy, decreased mobility for social interactions and, consequently, a deterioration in the perception of quality of life^(3)^. This is a pressing health problem for older adults and a major clinical and public health challenge in developing countries due to its high incidence, health-threatening effects, and the complications widely documented in the literature. Older adults often perceive falls negatively, interpreting them as a threat to their identity and autonomy, as they are commonly accompanied by physical and metaphysical repercussions^(4)^.

The factors that contribute to this process are related to intrinsic and/or extrinsic aspects that increase instability. Intrinsic factors include female sex, advanced age, comorbidities, especially musculoskeletal disorders, depression, and low self-efficacy in preventing falls. Extrinsic factors include uneven surfaces, slippery floors, inadequate lighting, and stairs without handrails^(5)^. Other elements are also added, such as the use of medications, previous history of falls, nutritional deficiencies, sedentary lifestyle, in addition to the relevance of the behavioral component, since both the most inactive and most active individuals have a greater risk of falls^(6)^.

In this context, healthcare professionals must be trained to identify older adults at high risk for falls. It is also crucial that they provide opportunities for information exchange with caregivers and older adults themselves, enabling health education that specifically addresses the risk factors specific to this population. These factors include physiological changes associated with aging, loss of muscle strength, reduced visual and auditory acuity, changes in balance and gait, decreased reflexes, chronic diseases, and polypharmacy^(7,8)^.

Given the above, and the clinical and social relevance of falls for older adults, it is undeniable that the increase in the number of older adults will likely require high healthcare costs due to the consequences of this event in this population. From this perspective, the guiding question for this study is: “What is the interface between the impact of falls and perceptions of quality of life among older adults?”.

## OBJECTIVES

To understand the relationship between falls and perceptions of quality of life in older adults.

## METHODS

### Ethical aspects

The study is part of a larger project entitled “*Promoção da saúde no processo de viver humano e prevenção de quedas, com vistas para o envelhecimento ativo de idosos do município de Itumbiara- Goiás (GO)*”. It was approved by the *Universidade Estadual de Goiás* Research Ethics Committee, in accordance with the guidelines of Resolution 466/2012 of the Brazilian National Health Council for research related to human beings. To preserve participant anonymity, alphanumeric codes were used, which made it possible to identify them with the letter “I” followed by numbers from 1 to 26 (I1, I2, I3... I26) corresponding to the order in which the interviews were conducted.

### Theoretical-methodological framework

Complexity theory was the theoretical framework used^(9)^.

### Study design

This is a qualitative study, using the COnsolidated criteria for REporting Qualitative research as a guide for the research process report.

### Study setting

The study was carried out in a Family Health Strategy (FHS), Itumbiara, Goiás, Brazil.

### Methodological procedures

Study participants were older adults living in the community of Itumbiara, Goiás, for over a year. The sample was intentionally selected using a convenience method, based on inclusion criteria. Forty-six older adults aged 60 and over, FHS users who had experienced at least one fall in the last year, were contacted in person by letter of invitation. Of these, 26 agreed to participate in the study and signed the Informed Consent Form.

### Data collection and organization

After participants agreed to participate in the study, dates and times were scheduled for interviews, based on participants’ availability, in a location that provided privacy. Twenty-six in-person interviews were conducted at the study site, each lasting an average of 30 minutes. Five pilot interviews were conducted, which were incorporated into the study because they significantly contributed to the proposed topic. No adjustments to the instrument were necessary. The interviews were conducted by a nurse responsible for FHS and two medical students, trained by the responsible advisor, who has expertise in qualitative research. Data collection took place from June to September 2024, through individual interviews guided by a semi-structured script, beginning with the triggering questions: have you ever experienced a fall after age 60? Tell me about it and its impact on your life. Tell me about the meaning of your experience. The interviews were audio-recorded and later transcribed and recorded in full by the researchers after validation by participants. No participant requested corrections, and the interviews were terminated based on the saturation criterion.

### Data analysis

The material was organized according to the three chronological poles of the thematic content analysis process, as proposed by Bardin (2011)^(10)^, namely: 1) pre-analysis; 2) material exploration; and 3) treatment of results, inference, and interpretation. In the “pre-analysis” stage, preparatory operations were carried out before the analysis itself, which consisted of the process of selecting documents or defining the *corpus*, delimiting the materials that were the objects of study; formulating the hypotheses and objectives of analysis, which guided the focus and guidelines of the analytical process; and developing the indicators that supported and supported the final interpretation of results.

The subsequent stage, called “material exploration”, refers to the process of systematically transforming raw data into analytical units. These units enabled an accurate and detailed description of the relevant characteristics of the content expressed in the text.

The third stage, called “treatment of results, inference, and interpretation”, aimed to highlight the information obtained from the analysis. To achieve this, quantification techniques were applied, ranging from simple methods, such as frequency, to more complex approaches, such as factor analysis. These procedures made it possible to organize and present the information clearly and visually, using diagrams, figures, models, and other resources to facilitate interpretation and communication of results.

## RESULTS

Data analysis revealed a predominantly female profile, accounting for 70.37% (n=19), married (95.59%), aged 63 to 88 years (85%), Catholic (55.56%), and retired (44.44%), ranging from one to 27 years. Regarding length of residence, most older adults, 79.92% (n=20), had lived in Itumbiara, Goiás, for several decades, with emphasis on those who had lived in this location since childhood or for more than 40 years, 88.46% (n=23).

Concerning experience with falls, 65.38% (n=17) reported experiencing them more frequently, meaning more than two falls. Furthermore, the home environment was the main location of falls, most often due to tripping, losing balance, or slipping.

The content analysis led to the thematic axis “*Impacts of falls on older adults living in the community*”, which encompassed four categories: 1) *Dynamics of falls, circumstances and associated factors;* 2) *Physical and emotional repercussions of falls;* 3) *Spirituality and social support network;* and 4) *Perceptions and suggestions for a safer life*.

### Dynamics of falls, circumstances and associated factors

This category presents how and where the fall occurred, as well as older adults’ experiences in this context.

The fall was often perceived as something natural, as if it were part of older adults’ lives, which reflects the perceived trivialization of an accident.

*I don’t think there’s any way to avoid falling*. (I7)

*I can’t say, because I don’t feel it’s possible to avoid fainting* [...]. *I’ve fallen 30 times.* (I12)

*Nothing changed after the fall; I was already retired*. (I16)

For some, public lighting, road and sidewalk maintenance, and continuous and adequate cleaning are seen as weak in terms of caring for public property and the risk of accidents, such as falls.

*The fall happened at night, when I was walking down the street* […]. *I slipped on some oil and fell.* (I11)

*I have cataracts. One day I was stepping off the sidewalk at night and ended up falling.* (I16)

*One of the falls I had, I tripped on the sidewalk and hit the ground face-first* [...]. (I17)

It is observed that most falls occurred in public and collective spaces, although the domestic environment has emerged as a particularly suitable place for this, since many residences are not adequately adapted to meet this audience’s needs.

*I slipped on the living room rug* (I1), *in the bathroom* (I9), *and in the dog’s pee.* (I10)

*I tripped going up the step to the living room door*. (I6)

From another perspective, some statements point to elements that permeate the social and utilitarian vision of older adults, with a consequent need to feel useful and active. It is worth noting that engaging in work activities, even informal ones, led to falls, as did situations such as climbing stairs or performing certain actions that put physical integrity at risk. This is intended to avoid the prejudice that older adults are synonymous with fragility and dependence.

*I fell three times from the aluminum ladder* […]. *At work.* (I4)

*I was cleaning the house and there was a small step, and I went to get the hose and fell* [...]. (I23)

### Physical and emotional repercussions of falls

Physical and emotional repercussions can be evidenced by impairments in health and functional capacity. This can lead to permanent physical or psychological consequences.

[...] *I was in pain and started walking slowly after the falls* [...]. (I4)

[...] *I had back pain and started taking pain injections.* (I15)

[...] *I can no longer do household chores, not even put my own food on the plate, due to shoulder pain.* (I25)

In this context, feelings of frustration and sadness emerged, mainly due to the perception of loss of autonomy and the difficulty in carrying out daily activities that were once considered simple.

[...] *I’m sad to see that I can’t perform simple daily activities.* (I12)

*I think I’m getting weaker, my legs hurt. They get so bad that I have to stop walking*. (I23)

Falls received greater attention from both older adults themselves and their families when they resulted in more serious consequences, such as the need for hospitalization and surgical intervention. On the other hand, many reported living with pain and constant exposure to the risk of further falls. Without fully considering their vulnerabilities and frailties, older adults remain susceptible to progressively more severe outcomes. It is also noted that older adults self-identify as “fallers” and accept the circumstances surrounding this event.

[…] *I’m more aware that at this age I can no longer perform any tasks I did when I was younger.* (I18)

*Accept that you’re getting older and that you have limitations. Accept using walking sticks and the assistance of people willing to help.* (I13)

The statements reveal nuances of the psycho-emotional and metaphysical realm. This aspect demonstrates the complexity and relevance of falls, as they can act as precursors to other traumas or conditions.

[…] *I was so scared of falling again and so sad that I still take medication for my headaches.* (I1)

[…] *we’re not the same people; we feel powerless. You lack confidence.* (I21)

### Spirituality and social support network

This category highlighted the relevance of spirituality in the post-fall recovery process and in coping with its consequences. Spirituality manifested itself as a central resource characterized by an attachment to the metaphysical. In this regard, God was reinterpreted as a trusted figure and replaced as a symbolic interlocutor to share experiences, fears, and difficulties. Spiritual practices, such as blessings and prayers, were also described as elements capable of promoting emotional comfort, hope, and resilience in the face of challenges.

[…] *although I’m not religious, I believe in God. I think no one can live well without having Someone to lean on.* (I10)

[…] *the physicians said I wouldn’t walk after the fall* […], *but with faith and God’s will, I was able to walk, and despite the problems, I have a certain freedom and autonomy*. (I20)

On the other hand, an older adult’s statement also revealed the worldview of someone who did not adhere to any religion and valued explanations based on evidence rather than beliefs. For them, coping with the consequences of a fall was likely associated with a search for practical solutions, such as health science interventions (nursing, medicine, physical therapy, among others) and adaptations to the home environment to prevent further falls. This approach emphasizes self-care, rationality, and control over tangible factors; after all, there was a prioritization of what can be proven and experienced in everyday life.

*Having had cataract surgery earlier could have prevented the fall* [...]. *I don’t think the spirituality aspect helped; I’m not a practitioner. I believe we should be more careful when walking down the street, paying attention*. (I16)

Social support plays an important role in maintaining older adults’ perceptions of quality of life, even when faced with changes in behavior or travel patterns. Reports show that, although they continue to carry out their daily activities, they have reduced the frequency of visits to places they used to frequent regularly, such as church, the market, and the supermarket. This decrease partly reflects a fear related to the possibility of further falls and physical insecurity, which can lead to gradual isolation.

[...] *I’m afraid of falling again.* (I5)

[...] *I’ve been avoiding walking outside for fear of falling.* (I13)

The presence of a social support network acts as a protective factor that helps mitigate psychological impacts and encourages the resumption of a more active routine. This applies both acutely, i.e., when falls occur and guidance is provided for necessary health treatments, and chronically, where direct encouragement, travel assistance, and emotional support are also provided. This support network strengthens older adults’ confidence and autonomy.

[...] *I even stopped going to my checkers games in the square.* (I1)

*I stopped leaving the house as much for fear of fainting alone on the street*. (I2)

### Perceptions and suggestions for a safer life

Older adults reflected on fall prevention factors that could have helped avoid this event, grouped into intrinsic and extrinsic factors as well as the acceptance of physical and functional limitations inherent to the aging process. Acceptance of limitations was a point emphasized, as they recognized the need to adjust behaviors and expectations to current conditions. For them, awareness of their own vulnerabilities and fragilities should not be seen as a loss, but as a step toward adopting safer practices and avoiding risky situations.

*Watch your step; balance declines with age.* (I3)

*Install grab bars and rugs in the bathroom to prevent falls. Remove rugs and reduce furniture to prevent tripping.* (I12)

Perceptions and suggestions for a safer life were expressed sometimes as a warning and learning experience, sometimes as resignation in the face of the consequences of a fall.

*Don’t have rugs in your home, and pay close attention to where you walk, especially in poorly lit areas.* (I11)

*Be careful and pay attention to what you’re doing, because I didn’t.* (I23)

Despite the context of naturalization and trivialization of falls, there were also manifestations from older adults about self-care, which, according to them, was related to the process of healthy living in the community, interactions and family arrangements.

*I had my husband remove the steps and build a ramp, both in the living room and at the kitchen door, which helped a lot.* (I5)

*Take supplements to strengthen your body* [...]. *Wear flat shoes that fit snugly.* (I18)

Safety was related to the difficulty in adequately perceiving the limitations imposed by the current condition of older adults, in contrast to their experiences in youth. Activities that were once routine and harmless now represented potential risks and required greater caution. This perspective highlights the need to adapt behaviors to minimize dangers in everyday life.

*I fell while chasing a calf to milk a cow* [...]. (I18)

*Be careful when cleaning ceramic floors* [...]. *Wear non-slip shoes and pay close attention when walking on the street. Avoid running.* (I20)

## DISCUSSION

Trivialization of falls can obscure the severity of the problem and cause older adults and society to underestimate the causes, consequences, and preventive measures. However, falls should be understood as a serious event capable of causing numerous repercussions and negative outcomes, which certainly affect older adults’ perceptions of quality of life, such as a greater risk of functional decline after the event. They also generate a greater risk of hospitalizations and associated complications, such as pressure injuries, as well as muscle immobility and atrophy, a burden on public coffers, a higher rate of morbidity and mortality, and an increase in vulnerability and fragility^(11)^.

Vulnerability in aging can have biological, socioeconomic, or psychosocial origins, with repercussions on multiple aspects of the human condition. Frailty, in turn, is a multifactorial syndrome characterized by dysregulation of the neuroendocrine system and immune system dysfunction. These factors result in manifestations such as weight loss, muscle weakness, low endurance, and reduced walking speed^(12)^.

Vulnerability can lead to the development of frailty in older adults. In this context, every frail older adult is considered vulnerable, but not all vulnerable older adults are frail. It is estimated that between 10% and 25% of the population over 65 is frail, a percentage that increases to approximately 45% in older adults aged 85 and older^(13)^. This vulnerability refers to a greater predisposition of the population to experience negative impacts from adverse conditions, since the natural aging process leads to a reduction in physiological reserves, making the body more susceptible to illness, falls, and infections. Furthermore, the presence of various chronic conditions, such as hypertension, diabetes, or arthritis, can exacerbate this vulnerability^(14)^.

Frailty is a clinical condition prevalent in older adults, characterized by reduced functional reserve and the body’s inability to cope with even low-intensity stressors, such as unintentional weight loss, fatigue, muscle weakness, slowness, and low physical activity levels. Frail older adults are more susceptible to adverse events, which are undesirable situations, whether expected or not, that occur during healthcare and result in any type of harm to patients in the biopsychosocial and spiritual spheres, such as falls and hospitalizations. The environment plays a fundamental role in frailty progression^(13,15)^.

Vulnerability and fragility are associated with an increased prevalence of disease and disability, resulting in a burden on families and increased healthcare costs^(16)^. Another significant aspect of vulnerability is social isolation, which can lead to emotional and psychological decline^(14)^.

Preserving functional capacity has a direct impact on older adults’ perceptions of quality of life, as specific limitations such as loss of strength, comorbidities, and falls lead to disabilities that compromise basic activities of daily living and instrumental activities of daily living. Thus, functional decline is directly related to increased frailty and the progressive development of disabilities, which, in turn, impairs the development of autonomy^(17)^.

It is estimated that by 2050, approximately one in three people aged 65 and older will experience at least one fall per year, and about half of these will result in injury. Both falls and fear of falling are common syndromes among older adults, with the potential to have serious consequences and significantly compromise their perceptions of quality of life^(18)^.

Fear of falling is characterized by anxiety when walking or excessive worry about the possibility of falling. This condition can reduce confidence in one’s ability to move, leading to depression, feelings of helplessness, social isolation, and behavioral changes. These factors affect functional mobility and promote physical dependence^(19)^. The prevalence of fear of falling ranges from 20% to 85% among non-institutionalized older adults and is associated with poorer health conditions, advanced age, depression, difficulties in daily activities, injuries caused by falls, decreased social interaction, and a sedentary lifestyle^(20)^. These aspects highlight the importance of interventions that encourage the development of support networks, promote older adults’ confidence in their autonomy, and strengthen awareness of preventive measures^(11)^.

The findings of this study are corroborated by the contributions of some authors^(21,22)^ who indicate the home as the main scenario for falls, followed by public collective environments. However, these episodes can be significantly reduced through prevention and health promotion measures. This highlights the importance of assessing and adapting the home environment, with the aim of making it safer and more functional, and investing in space adaptations, such as installing grab bars, removing loose rugs and improving lighting.

Falls in public spaces are indicative of structural weaknesses such as street lighting, poorly maintained sidewalks, and insufficient cleaning. These problems point to neglect in both public space care and accident prevention. Urban maintenance and planning play an essential role in everyone’s safety, but especially in protecting the most vulnerable groups. Furthermore, it’s important to consider conditions such as syncope, labyrinth disorders, and heart problems-intrinsic factors common in older adults.

Syncope is a transient loss of consciousness caused by a temporary reduction in cerebral blood flow and, therefore, a significant factor in falls in older adults. This event can contribute to fear and loss of the prospect of autonomy and confidence in being alone, regardless of location or environment. The causes of syncope can vary and include autonomic dysfunction, orthostatic hypotension, among other common conditions in older adults^(23)^.

Labyrinthopathies are disorders that affect the labyrinth and can cause dizziness, vertigo, and imbalance, often associated with difficulty performing daily tasks that require motor coordination. In older adults, they tend to be more prevalent due to the natural aging of the vestibular system and the higher incidence of chronic diseases that can compromise its function^(24)^.

Heart problems also play a significant role in the genesis of falls. Conditions such as bradycardia can reduce cerebral blood flow and lead to episodes of dizziness and fainting. Atrioventricular block is another factor that can result in significant bradycardia and a consequent risk of falls^(23)^.

Aging is also associated with factors such as sarcopenia, osteoporosis, and decreased sensory capacity, which exacerbate the effects of these conditions. The presence of multimorbidity and polypharmacy, frequently observed in older adults, increases the risk of falls^(25)^.

The ideal conditions for actions, interventions, and fall prevention in older adults include the identification and appropriate management of intrinsic and extrinsic factors. This involves regular clinical assessment, treatment of underlying conditions, monitoring cardiac conditions, and promoting activities that improve balance, muscle strength, and flexibility. It is also important to consider installing adequate lighting, non-slip flooring, seats with barriers for greater safety during bathing, installing grab bars, carefully organizing spaces, and removing loose rugs^(26)^. Falls in older adults are often associated with factors such as steps, uneven surfaces, rugs, pets, and objects on the floor^(27)^. Therefore, by adopting practices such as reorganizing environments, providing educational guidance, and adapting physical space, it is possible to minimize the risk of accidents due to external causes, including falls.

Among the older adults surveyed, falls were related to a search for autonomy and usefulness. A social perception emerged that associates aging with fragility and dependence, encouraging them to remain active, even in risky situations. From performing a simple household task, older adults often challenge their own physical limits, perhaps in an attempt to resist prejudice and assert their own independence and autonomy.

In this context, guidance is therefore needed to prevent this event in this population, with an emphasis on extrinsic factors, such as adapting the home, to ensure a safe environment^(28)^. It is also imperative to guide older adults and their families about the risks and consequences, through health promotion, in order to correct or minimize risk factors.

### Study limitations

Although the study has the potential to be replicated and expanded in other FHSs, only older adults from one FHS were surveyed.

### Contributions to nursing, health, or public policy

Understanding the relationship between falls among older adults in the community and their perceptions of quality of life enables the adoption of preventive measures, both individual and collective, which will potentially impact their fragility and vulnerability and reduce hospitalizations and unfavorable outcomes, such as irreparable sequelae and death. Analyzing these factors, based on the reality of older adults living in the community, can promote comprehensive, comprehensive, and more effective care at all levels of prevention. These strategies reinforce the implementation, deployment, and maintenance of older adults’ autonomy and enable family and social interaction, which prompts reflections on prevention-oriented practices.

## FINAL CONSIDERATIONS

The relationship between falls in older adults and perceptions of quality of life is complex, as this event directly impacts physical and emotional health as well as accentuating the fragility and vulnerability of this population group.

Many older adults tend to naturalize this occurrence, influenced by social perspectives that associate aging with the inevitability of vulnerability and fragility. This view reinforces the need for strategies that promote awareness of the risks and consequences of falls, while also encouraging prevention and care practices. Valuing social support networks and spirituality has proven to be relevant for coping with the repercussions of falls and is an essential resource in the pursuit of better perceptions of quality of life.

In this context, healthcare professionals are encouraged to produce knowledge targeted at older adults through research on interventions to prevent this event, enabling them to manage fragility and vulnerability.

## Data Availability

The research data are available within the article.

## References

[1] Paula NF, Santos SMA. (2015). Epidemiology of accidental falls among the elderly: survey of the period 2003-2012. REME Rev Min Enferm.

[2] Lee HJ, Yun J. (2020). Health-related quality of life in South Korean community-dwelling older adults with multimorbidity: a convergent parallel mixed-methods approach. Qual Life Res.

[3] Silva TL, Motta VV, Garcia WJ, Arreguy-Sena C, Pinto PF, Parreira PMSD (2021). Quality of life and falls in elderly people: a mixed methods study. Rev Bras Enferm.

[4] Sena AC, Alvarez AM, Nunes SFL, Costa NPS. (2021). Nursing care related to fall prevention among hospitalized elderly people: an integrative review. Rev Bras Enferm.

[5] Andrade CLF, Moreira NFC, Barcelos IO, Rodrigues JC, Alves KRS, Andrade DF (2024). Envelhecer e as principais síndromes geriátricas: relação entre fragilidade, incontinência urinária e quedas. REAS.

[6] Andrade RC, Santos MM, Ribeiro EE, Santos JDO, Campos HLM, Leon EB. (2024). Polypharmacy, potentially inappropriate medications, and the vulnerability of older adults. Rev Bras Geriatr Gerontol.

[7] Bernardes RS, Barros RS, Silva FS, Pequeno SA, Alves AT, Garcia PA. (2024). Urinary symptoms, falls and fear of falling in older people with cognitive impairment. Fisioter Mov.

[8] Sá GGM, Santos AMR, Galindo NM, Carvalho KM, Feitosa CDA, Mendes PN. (2020). Building and validating an educational video for elderly individuals about fall risks. Rev Bras Enferm.

[9] Morin E. (2011). Introdução ao pensamento complexo.

[10] Bardin L. (2011). Análise de conteúdo.

[11] Luzardo AR, Paula NF, Medeiros M, Wolkers PCB, Santos SMA. (2018). Repercussions of hospitalization due to fall of the elderly: health care and prevention. Rev Bras Enferm.

[12] Santos JAD, Santos SM, Paulino MD, Carneiro JA, Costa FM. (2024). Prevalence and factors associated with frailty in older adults with hypertension using the Edmonton Frail Scale and the Clinical Functional Vulnerability Index-20. Rev Bras Geriatr Gerontol.

[13] Jesus IT, Orlandi AA, Grazziano ES, Zazzetta MS. (2017). Frailty of the socially vulnerable elderly. Acta Paul Enferm.

[14] Sousa CR, Coutinho JFV, Freire JB, Barbosa RGB, Marques MB, Diniz JL. (2022). Factors associated with vulnerability and fragility in the elderly: a cross-sectional study. Rev Bras Enferm.

[15] Andrade CLF, Moreira NFC, Barcelos IO, Palheta WJN, Oliveira RH, Ferreira JD (2024). Desafios e estratégias de segurança do paciente no centro cirúrgico: importância da atuação do(a) enfermeiro(a). CLCS.

[16] Carlos AG, Dias VN, Perracini MR, Doná F, Sousa AGP, Gazzola JM. (2024). Postural balance and associated factors with the fall risk assessed in older adults with type 2 diabetes mellitus. Rev Bras Geriatr Gerontol.

[17] Freitas FFQ, Soares SM. (2019). Clinical-functional vulnerability index and the dimensions of functionality in the elderly person. Rev Rene.

[18] Friedman SM, Munoz B, West SK, Rubin GS, Fried LP. (2002). Falls and fear of falling: which comes first? a longitudinal prediction model suggests strategies for primary and secondary prevention. J Am Geriatr Soc.

[19] Freiberger E, Vreede P. (2011). Falls recall: limitations of the most used inclusion criteria. Eur Rev Aging Phys Act.

[20] Zijlstra GAR, Haastregt JCM, Eijk JTM, Rossum E, Stalenhoef PA, Kempen GIJM. (2007). Prevalence and correlates of fear of falling, and associated avoidance of activity in the general population of community-living older people. Age Ageing.

[21] Fonseca RFMR, Matumoto S. (2020). Falls prevention in elderly: what official Brazilian publications say?. J Nurs Health.

[22] Oliveira SRN, Messias FML, Cândido JAB, Torres GMC, Figueiredo IDT, Pinto AGA (2021). Factors associated with falls in older adults: a household survey. Rev Bras Promoç Saúde.

[23] Silva EMFL, Barbosa MT, Miranda CES. (2015). Syncope in the elderly. Rev Méd Minas Gerais.

[24] Maranhão ASA, Godofredoc VR, Penidoa NO. (2016). Suppurative labyrinthitis associated with otitis media: 26 years’ experience. Braz J Otorhinolaryngol.

[25] Morsch P, Myskiw M, Myskiw JC. (2016). Falls’ problematization and risk factors identification through older adults’ narrative. Ciênc Saúde Colet.

[26] Duarte GP, Santos JLF, Lebrão ML, Duarte YAO. (2019). Relationship of falls among the elderly and frailty componentes. Rev Bras Epidemiol.

[27] Galvan SS, Santos CB, Doring M, Portella MR. (2017). Prevalence of household falls in long-lived adults and association with extrinsic factors. Rev Latino-Am Enfermagem.

[28] Maia JC, Diniz JL, Sousa CR, Oliveira FGL, Evangelista BP, Coutinho JFV (2023). Interactive gerontechnology for fall prevention in the elderly: a descriptive study. Rev Bras Enferm.

