# Supplementary material for: Interface between falls and perceptions of quality of life of older adults: a qualitative study
Source: Rev Bras Enferm. 2025 Dec 8;78(6):e20250007. doi: 10.1590/0034-7167-2025-0007 (PMC12707524; doi:10.1590/0034-7167-2025-0007)
Supplement: Translation

# Translation: Interface entre quedas e percepções de qualidade de vida de idosos: estudo qualitativo

**DOI:** 10.1590/0034-7167-2025-0007pt

**Authors:** Caio Leonardo Faria Andrade, Patrícia Chaves da Silva, Kevin Silva Moreira, Jonathan Ballico de Moraes, Juliana Balbinot Reis Girondi, Newton Ferreira de Paula

**Affiliations:** I Fundação Presidente Antônio Carlos. Uberlândia, Minas Gerais, Brasil; II Universidade Estadual de Goiás. Itumbiara, Goiás, Brasil; III Universidade Federal de Santa Catarina. Florianópolis, Santa Catarina, Brasil

**Keywords:** Acidentes por Quedas, Qualidade de Vida, Idoso, Fragilidade, Vulnerabilidade.

## Abstract

**Objetivos:**

compreender a relação entre quedas e percepções de qualidade de vida em idosos.

**Métodos:**

estudo descritivo e exploratório, de abordagem qualitativa, realizado com idosos em Estratégia de Saúde da Família de Itumbiara, Goiás, por meio da Análise de Conteúdo de Bardin.

**Resultados:**

a análise das entrevistas com 26 idosos revelou quatro eixos temáticos: *Dinâmica das quedas, circunstâncias e fatores associados; Repercussões físicas e emocionais das quedas; Espiritualidade e rede de suporte social;* e *Percepções e sugestões para uma vida mais segura*. As quedas potencializam a fragilidade e a vulnerabilidade de idosos, afetam a saúde, dependência e capacidade funcional e tendem a ser naturalizadas e banalizadas pelos próprios idosos.

**Considerações Finais:**

as quedas impactam a saúde física e emocional dos idosos. É necessário sensibilizá-los acerca das causas, consequências e prevenção deste evento, no intuito de promover um envelhecimento saudável e ativo.

## INTRODUÇÃO

Os idosos estão em processo contínuo de desenvolvimento biopsicossocioespiritual, enriquecidos por uma considerável experiência de vida. Embora alterações fisiológicas próprias do envelhecimento não configurem necessariamente doenças, podem aumentar a fragilidade e vulnerabilidade a algumas moléstias. Por outro lado, muitos idosos chegam à velhice com Doenças Crônicas Não Transmissíveis (DCNTs) adquiridas ao longo da vida adulta, o que eleva a fragilidade e o risco de experimentarem, por exemplo, as quedas^(1)^.

A queda pode ser compreendida como o contato não intencional com o solo ou outra superfície de apoio, resultante da mudança de posição do indivíduo para nível inferior ao que se encontra no momento anterior a este evento^(2)^. Pode resultar em redução da capacidade funcional, comprometimento da autonomia, diminuição da mobilidade para interações sociais e, consequentemente, deterioração da percepção de qualidade de vida^(3)^. Trata-se de um problema iminente para a saúde dos idosos e um dos principais desafios clínicos e de saúde pública nos países em desenvolvimento, devido à alta incidência, por comprometer o estado de saúde e gerar complicações amplamente documentadas na literatura. Frequentemente, os idosos percebem as quedas de forma negativa, interpretando-as como uma ameaça à identidade e autonomia, uma vez que comumente são acompanhadas de repercussões físicas e metafísicas^(4)^.

Os fatores que contribuem nesse processo estão relacionados a aspectos intrínsecos e/ou extrínsecos que aumentam a instabilidade. Entre os fatores intrínsecos, destacam-se: sexo feminino, idade avançada, comorbidades, especialmente as doenças osteomusculares, depressão e baixa autoeficácia para evitar quedas. Já os fatores extrínsecos incluem superfícies irregulares, pisos escorregadios, iluminação inadequada e escadas sem corrimão^(5)^. Somam-se ainda outros elementos, como o uso de medicamentos, histórico prévio de quedas, deficiências nutricionais, sedentarismo, além da relevância do componente comportamental, visto que tanto os indivíduos mais inativos como os mais ativos possuem maior risco de quedas^(6)^.

Nesse contexto, os profissionais de saúde devem estar capacitados para identificar idosos com elevada suscetibilidade a quedas. Mostra-se fundamental também que oportunizem a troca de informações com os cuidadores e com os próprios idosos, com o fito de possibilitar o processo de educação em saúde que aborda, especialmente, os fatores de risco específicos dessa população. Tais componentes incluem alterações fisiológicas do envelhecimento, perda de força muscular, redução da acuidade visual e auditiva, alterações no equilíbrio e marcha, diminuição dos reflexos, doenças crônicas e polifarmácia^(7,8)^.

Diante do exposto, e dada a relevância clínica e social das quedas para a população idosa, é inegável que o aumento no número de idosos, possivelmente, demandará gastos elevados com tratamentos de saúde, em virtude das consequências causadas por este evento nessa população. Sob esta perspectiva, tem-se como questão norteadora para o desenvolvimento deste estudo: “Como se apresenta a interface entre o impacto do evento queda e as percepções de qualidade de vida na população de idosos?”.

## OBJETIVOS

Compreender a relação entre quedas e percepções de qualidade de vida em idosos.

## MÉTODOS

### Aspectos éticos

O estudo é parte de projeto maior intitulado “Promoção da saúde no processo de viver humano e prevenção de quedas, com vistas para o envelhecimento ativo de idosos do município de Itumbiara- Goiás (GO)”. Foi aprovado pelo Comitê de Ética em Pesquisa com Seres Humanos da Universidade Estadual de Goiás (CEPSH/UEG), em conformidade com as diretrizes da Resolução nº 466/2012 do Conselho Nacional de Saúde para pesquisas relacionadas a seres humanos. Para preservar o anonimato dos participantes, foram utilizados códigos alfanuméricos, o que possibilitou identificá-los com a letra “I” seguida de números de 1 a 26 (I1, I2, I3... I26) correspondentes à ordem de realização das entrevistas.

### Referencial teórico-metodológico

A teoria da complexidade foi o referencial teórico utilizado^(9)^.

### Tipo de estudo

Estudo qualitativo, com utilização do Consolidated Criteria for Reporting Qualitative Research (COREQ) como norteador do relatório do processo da pesquisa.

### Cenário do estudo

Estratégia de Saúde da Família, Itumbiara, Goiás, Brasil.

### Procedimentos metodológicos

Os participantes do estudo foram idosos residentes na comunidade de Itumbiara/GO há mais de um ano. A seleção da amostra foi intencional, por método de conveniência, mediante critérios de inclusão. Realizou-se contato presencial, por meio de carta-convite a 46 idosos com 60 anos e mais, usuários da Estratégia de Saúde da Família (ESF) que já vivenciaram, pelo menos, um episódio de queda no último ano. Destes, 26 aceitaram fazer parte da pesquisa e assinaram o Termo de Consentimento Livre e Esclarecido (TCLE).

### Coleta e organização dos dados

Após o aceite em participar da pesquisa, foram marcados dias e horários para as entrevistas, conforme disponibilidade dos participantes, em local que possibilitasse a privacidade. Foram realizadas 26 entrevistas, presenciais, no local do estudo, com duração média de 30 minutos cada. Foram realizadas cinco entrevistas-piloto, as quais foram incorporadas ao estudo por contribuírem sobremaneira com a temática proposta. Não houve necessidade de ajustes no instrumento. As entrevistas foram conduzidas por uma enfermeira responsável pela Estratégia de Saúde da Família e por dois graduandos em medicina, treinados pelo orientador responsável, que possui expertise em pesquisa qualitativa. O período de coleta dos dados ocorreu de junho a setembro de 2024, por meio de entrevista individual, guiada por um roteiro semiestruturado, iniciado pelas perguntas disparadoras: Você já experienciou alguma queda após os 60 anos? Conte-me como foi e as repercussões em sua vida. Fale-me sobre os significados da sua experiência. As entrevistas foram audiogravadas, posteriormente transcritas e registradas integralmente pelos pesquisadores, após validação dos participantes. Nenhum participante solicitou correção, e as entrevistas foram encerradas pelo critério de saturação.

### Análise dos dados

O material foi organizado de acordo com os três polos cronológicos do processo de Análise de Conteúdo Temática, conforme proposto por Bardin (2011)^(10)^, a saber: 1) pré-análise; 2) exploração do material; e 3) tratamento dos resultados, inferência e interpretação. Na etapa “pré-análise”, foram realizadas operações preparatórias antes da análise propriamente dita, que consistiu no processo de seleção dos documentos ou definição do *corpus*, com delimitação dos materiais que foram objetos de estudo; formulação das hipóteses e dos objetivos da análise, que orientaram o foco e as diretrizes do processo analítico; e elaboração dos indicadores que sustentaram e embasaram a interpretação final dos resultados.

A etapa subsequente, denominada “exploração do material”, refere-se ao processo de transformação sistemática dos dados brutos em unidades analíticas. Essas unidades possibilitaram uma descrição precisa e detalhada das características relevantes do conteúdo expresso no texto.

A terceira etapa, denominada “tratamento dos resultados, inferência e interpretação”, objetivou destacar as informações obtidas na análise. Para isso, foram aplicadas técnicas de quantificação que variaram de métodos simples, como frequência, até abordagens mais complexas, como a análise fatorial. Esses procedimentos possibilitaram organizar e apresentar as informações de maneira clara e visual, por meio de diagramas, figuras, modelos e outros recursos facilitadores da interpretação e da comunicação dos resultados.

## RESULTADOS

A análise dos dados revelou um perfil com predominância do gênero feminino, que representou 70,37% (n=19), casadas (95,59%), na faixa etária de 63 a 88 anos (85%), católicas (55,56%) e aposentadas (44,44%), com variação de um a 27 anos. Em relação ao tempo de residência, a maior parte dos idosos, 79,92% (n=20), residia em Itumbiara-GO há várias décadas, com destaque para aqueles residentes neste local desde a infância ou há mais de 40 anos, 88,46% (n=23).

Em relação à experiência com quedas, 65,38% (n=17) relataram ter experimentado o evento com maior frequência, ou seja, mais de duas quedas. Além disso, o ambiente doméstico foi o principal local de ocorrência, a maioria por tropeços, desequilíbrios e resvalos.

Da análise dos conteúdos emergiu o eixo temático “*Repercussões das quedas em idosos que vivem em comunidade*”, o qual reuniu quatro categorias: 1) *Dinâmica das quedas, circunstâncias e fatores associados*; 2) *Repercussões físicas e emocionais das quedas*; 3) *Espiritualidade e rede de suporte social*; e 4) *Percepções e sugestões para uma vida mais segura*.

### Dinâmica das quedas, circunstâncias e fatores associados

Nessa categoria são apresentados como e onde o evento queda ocorreu e as experiências vivenciadas pelos idosos nesse contexto.

O evento queda foi frequentemente percebido como algo natural, como se fizesse parte da vida dos idosos, o que denota a percepção de banalização de um acidente.

*Acredito não ter como evitar a queda.* (I7)

*Não sei dizer, pois não sinto ser possível evitar os desmaios* […]. *Já caí 30 vezes*. (I12)

*Não mudou nada após a queda, eu já estava aposentado*. (I16)

Para alguns, iluminação pública, manutenção de vias e calçadas, além da limpeza contínua e adequada, mostram-se fragilizadas naquilo que se refere ao cuidado com o bem público e ao risco de acidentes, como o evento queda.

*A queda ocorreu à noite, quando estava andando na rua* […]. *Escorreguei em óleo e caí.* (I11)

*Eu tenho catarata. Um dia fui descer da calçada de noite e acabei caindo*. (I16)

*Uma das quedas que tive tropecei na calçada e bati de cara no chão* [...]. (I17)

Observa-se que as quedas, em sua maioria, ocorreram em espaços públicos e coletivos, embora o ambiente doméstico tenha emergido como um local especialmente propício para tal, uma vez que muitas residências não estão adequadamente adaptadas para atender às necessidades desse público.

*Escorreguei no tapete da sala* (I1), *no banheiro* (I9) e *no xixi do cachorro*. (I10)

*Eu tropecei ao subir o degrau da porta da sala*. (I6)

Sob outra perspectiva, alguns depoimentos apontam para elementos que permeiam a visão social e utilitária da pessoa idosa, com consequente necessidade de se sentirem úteis e ativos. Vale ressaltar que o exercício de atividade laboral, mesmo que informal, acarretou quedas, assim como situações tais como subir uma escada ou executar determinada ação que coloque em risco a integridade física, com o fito de evitar o preconceito de que pessoa idosa é sinônimo de fragilidade e dependência.

*Tive três quedas da escada de alumínio* […]. *Trabalhando*. (I4)

*Estava limpando a casa e tem um degrau pequeno, e eu fui pegar a mangueira e caí* [...]. (I23)

### Repercussões físicas e emocionais das quedas

Repercussões físicas e emocionais podem ser evidenciadas por meio da constatação de prejuízos na condição de saúde e na capacidade funcional. Isso gerou consequências de caráter permanente, físicas ou psicológicas.

[…] *Fiquei com dor e passei a andar devagar depois das quedas* [...]. (I4)

[…] *Fiquei com dor na coluna e comecei a tomar injeção para dor*. (I15)

[…] *Não consigo mais realizar atividades domésticas, nem ao menos colocar a própria comida no prato, devido às dores no ombro*. (I25)

Nesse contexto, emergiram sentimentos de frustração e tristeza, principalmente pelas percepções de perda de autonomia e pela dificuldade em realizar atividades cotidianas outrora consideradas simples.

[...] *triste por ver que não consigo realizar atividades simples do dia a dia*. (I12)

*Acho que venho enfraquecendo, minhas pernas doem. Ficam ruins, aí tenho que parar de andar*. (I23)

As quedas receberam maior atenção tanto dos próprios idosos quanto de seus familiares no instante em que resultaram em consequências mais graves, como a necessidade de hospitalização e intervenção cirúrgica. Por outro lado, muitos relataram conviver com a dor e com a constante exposição ao risco de novas quedas. Sem considerar plenamente suas vulnerabilidades e fragilidades, os idosos permanecem suscetíveis a desfechos progressivamente mais severos. Nota-se, ainda, a autoidentificação do idoso como “caidor” e a aceitação das circunstâncias que permeiam este evento.

[…] *Tenho mais noção de que nesta idade já não consigo desempenhar qualquer função que fazia quando mais jovem*. (I18)

*Aceite que está envelhecendo, e que possui limitações. Aceite usar bengalas e o auxílio de pessoas dispostas a ajudar.* (I13)

Os relatos revelam nuances do campo psicoemocional e metafísico. Esse aspecto demonstra a complexidade e relevância das quedas, uma vez que podem agir como precursoras de outros traumas ou afecções.

[…] *Fiquei com muito medo de cair novamente e uma tristeza, que tomo remédio até hoje pra cabeça.* (I1)

[…] *A gente não é a mesma pessoa, se sente impotente. Você não tem confiança.* (I21)

### Espiritualidade e rede de suporte social

Nessa categoria foi evidenciada a relevância da espiritualidade no processo de recuperação no pós-queda e nos enfrentamentos das consequências. A espiritualidade manifestou-se como um recurso central caracterizado pelo apego ao metafísico. Nesse sentido, Deus foi ressignificado como uma figura de confiança e ocupou o lugar de um interlocutor simbólico para compartilhar experiências, medos e dificuldades. Práticas espirituais, como bênçãos e orações, também foram descritas como elementos capazes de promover conforto emocional, esperança e resiliência diante dos desafios vivenciados.

[…] *Apesar de não possuir religião, acredito em Deus. Penso que ninguém consegue viver bem sem ter Alguém a se apoiar.* (I10)

[…] *Os médicos diziam que eu não andaria após a queda* […], *mas, com a fé e a vontade de Deus, consegui andar e, apesar dos problemas, tenho certa liberdade e autonomia*. (I20)

Por outro lado, também emergiu, no depoimento de um idoso, a visão de mundo de alguém não adepto a qualquer religião, que valorizava explicações baseadas em evidências e não em crenças. Para ele, o enfrentamento das consequências da queda possivelmente estava associado a uma busca por soluções práticas, como intervenções de ciências da saúde (enfermagem, medicina, fisioterapia, entre outros) e adaptações no ambiente doméstico a fim de prevenir novas quedas. Essa abordagem enfatiza o autocuidado, a racionalidade e o controle acerca dos fatores tangíveis, afinal houve priorização do que pode ser comprovado e experimentado no cotidiano.

*Ter feito a cirurgia de catarata antes poderia ter evitado a queda* [...]*. Não acho que a questão da espiritualidade ajudou, não sou praticante. Creio que devemos ter mais cuidado quando formos andar na rua, prestando atenção*. (I16)

O suporte social desempenha papel importante na manutenção das percepções da qualidade de vida dos idosos, mesmo diante de alterações de comportamento ou no padrão de deslocamento. Os relatos mostram que, apesar de continuarem a realizar suas atividades de vida cotidianas, reduziram a frequência de visitas a locais que costumavam frequentar regularmente, como igreja, feira e supermercado. Essa diminuição reflete, em parte, um receio relacionado à possibilidade de novas quedas e à insegurança física, o que pode ocasionar isolamento gradual.

[…] *Fico com medo de cair novamente.* (I5)

[…] *Tenho evitado andar na rua, por medo de queda.* (I13)

A presença de uma rede de apoio social funciona como um fator de proteção que auxilia a mitigar os impactos psicológicos e a incentivar a retomada de uma rotina mais ativa. Isso é válido tanto em nível agudo, ou seja, no momento da ocorrência de quedas e no direcionamento para as tratativas de saúde que se fizeram necessárias, quanto no nível crônico, onde há também, por meio de incentivo direto, o acompanhamento em deslocamento e apoio emocional. Esta rede de suporte fortalece a confiança e a autonomia dos idosos.

[…] *Deixei até de frequentar os meus jogos de dama na praça*. (I1)

*Parei de sair tanto de casa por medo de ocorrer a síncope sozinha na rua*. (I2)

### Percepções e sugestões para uma vida mais segura

Os idosos refletiram acerca dos fatores de prevenção de quedas que poderiam ter auxiliado a evitar este evento, agrupados em fatores intrínsecos e extrínsecos, além da aceitação das limitações físicas e funcionais inerentes ao processo de envelhecimento. A aceitação das limitações foi um ponto enfatizado, pois reconheceram a necessidade de ajustar comportamentos e expectativas às condições atuais. Para eles, a conscientização acerca das próprias vulnerabilidades e fragilidades não deve ser vista como uma perda, mas como um passo para adotar práticas mais seguras e evitar situações de risco.

*Olhe bem onde pisa, o equilíbrio diminui com a idade.* (I3)

*Coloque barras de apoio e tapetes no local de banho para evitar quedas. Tire tapetes, e diminua móveis para evitar tropeçar.* (I12)

As percepções e sugestões para uma vida mais segura foram expressas ora como alerta e aprendizado, ora como resignação diante da consequência da queda.

*Não tenha tapetes em casa, e preste muita atenção ao andar, principalmente em locais com pouca iluminação.* (I11)

*Tenha cuidado e preste atenção no que está fazendo, porque eu não prestei.* (I23)

Apesar do contexto de naturalização e banalização das quedas, houve também manifestações dos idosos acerca do autocuidado, o qual, segundo eles, estava relacionado ao processo de viver saudável em comunidade, às interações e aos arranjos familiares.

*Fiz meu marido tirar os degraus e fazer rampa, tanto na sala quanto na porta da cozinha, que ajudou muito*. (I5)

*Tome suplementos para fortalecer o corpo* […]. *Use sapato rasteiro, que se prende bem ao pé.* (I18)

A segurança foi relacionada à dificuldade em perceber adequadamente as limitações impostas pela condição atual de pessoa idosa, em contraste com a vivência na juventude. Atividades outrora rotineiras e inofensivas agora representavam potenciais riscos e exigiam maior cautela. Essa perspectiva evidencia a necessidade de adaptar comportamentos para minimizar perigos no cotidiano.

*Caí correndo atrás de bezerro para tirar leite de vaca* [...]. (I18)

*Cuidado ao lavar pisos de cerâmica* […]. *Use calçados antiderrapantes e preste muita atenção quando andar na rua. Evite correr.* (I20)

## DISCUSSÃO

A banalização do evento queda pode obscurecer a gravidade do problema e fazer com que os próprios idosos e a sociedade não deem a devida importância às causas, consequências e medidas preventivas. No entanto, as quedas devem ser compreendidas como um evento que precisa ser reconhecido como algo sério e capaz de ocasionar inúmeras repercussões e desfechos negativos, os quais, certamente, afetam as percepções de qualidade de vida das pessoas idosas, como maior risco de declínio funcional após o evento. Geram, também, maior risco de hospitalizações e complicações associadas, como lesão por pressão, além de imobilidade e atrofia muscular, oneração dos cofres públicos, maior taxa de morbimortalidade e potencialização da vulnerabilidade e fragilidade^(11)^.

A vulnerabilidade no envelhecimento pode ter origem biológica, socioeconômica ou psicossocial, com repercussões em múltiplos aspectos da condição humana. A fragilidade, por sua vez, é uma síndrome multifatorial caracterizada pela desregulação do sistema neuroendócrino e disfunções no sistema imunológico. Esses fatores resultam em manifestações como perda de peso, fraqueza muscular, baixa resistência e redução da velocidade ao caminhar^(12)^.

A vulnerabilidade pode levar ao desenvolvimento da fragilidade em idosos. Nesse contexto, todo idoso frágil é considerado vulnerável, mas nem todos os idosos em situação de vulnerabilidade são frágeis. Estima-se que entre 10% e 25% da população com mais de 65 anos esteja em condição de fragilidade, percentual que aumenta para cerca de 45% em idosos com 85 anos ou mais^(13)^. Essa vulnerabilidade refere-se a uma maior predisposição da população a experimentar impactos negativos diante de condições adversas, uma vez que o processo natural de envelhecimento acarreta a redução das reservas fisiológicas, o que torna o organismo mais suscetível a doenças, quedas e infecções. Além disso, a presença de várias condições crônicas, como hipertensão, diabetes ou artrite, pode agravar essa vulnerabilidade^(14)^.

Já a fragilidade é uma condição clínica presente de maneira intensa nos idosos, caracterizada pela redução da reserva funcional e pela incapacidade do organismo de lidar com estressores, mesmo que de baixa intensidade, tais como perda de peso não intencional, fadiga, fraqueza muscular, lentidão e baixo nível de atividade física. Nesse sentido, idosos frágeis são mais suscetíveis a eventos adversos, que são situações indesejadas, esperadas ou não, que ocorrem durante a assistência à saúde e resultam em qualquer tipo de dano ao paciente na esfera biopsicossocioespiritual, como quedas e hospitalizações. O ambiente desempenha papel fundamental na progressão da fragilidade^(13,15)^.

A vulnerabilidade e a fragilidade estão associadas ao aumento da prevalência de doenças e incapacidades, com consequente sobrecarga para as famílias e elevação dos custos nos serviços de saúde^(16)^. Outro aspecto significativo da vulnerabilidade é o isolamento social, pela possiblidade de causar declínio emocional e psicológico^(14)^.

A preservação da capacidade funcional tem impacto direto nas percepções de qualidade de vida dos idosos, pois limitações específicas de perda de força, comorbidades e quedas levam a incapacidades que comprometem as Atividades Básicas de Vida Diária (ABVDs) e Atividades Instrumentais de Vida Diária (AIVDs). Dessa forma, o declínio funcional está diretamente relacionado ao aumento da fragilidade e ao desenvolvimento progressivo de incapacidades, o que, por sua vez, prejudica o desenvolvimento da autonomia^(17)^.

Estima-se que, em 2050, aproximadamente uma em cada três pessoas com 65 anos ou mais experimente ao menos uma queda por ano, e cerca da metade destas resultem em lesões. Tanto as quedas quanto o medo de cair são síndromes frequentes entre os idosos, com potencial para gerar consequências graves e comprometer significativamente suas percepções de qualidade de vida^(18)^.

O medo de cair é caracterizado pela ansiedade ao caminhar ou pela preocupação excessiva com a possibilidade de queda. Essa condição pode reduzir a confiança na própria capacidade de locomoção, levar a quadros de depressão, sentimentos de desamparo, isolamento social e alterações comportamentais. Esses fatores afetam a mobilidade funcional e promovem a dependência física^(19)^. A prevalência do medo do evento queda varia de 20% a 85% entre idosos não institucionalizados e está associado a piores condições de saúde, idade avançada, depressão, dificuldades nas AVDs, lesões causadas por quedas, diminuição do convívio social e estilo de vida sedentário^(20)^. Esses aspectos alertam para a importância de intervenções que estimulem a construção de redes de suporte, promovam a confiança do idoso em sua autonomia e fortaleçam a sensibilização acerca de medidas preventivas^(11)^.

Corroboram os achados desta pesquisa as contribuições de alguns autores^(21,22)^ que indicam o domicílio como principal cenário de ocorrência de quedas, seguido de ambientes públicos coletivos. Contudo, esses episódios podem ser significativamente reduzidos por meio de medidas de prevenção e promoção da saúde. Isso ressalta a importância de avaliar e adaptar o ambiente doméstico, com o objetivo de torná-lo mais seguro e funcional, e investir nas adequações dos espaços, como a instalação de barras de apoio, remoção de tapetes soltos e melhoria na iluminação.

As quedas em ambientes coletivos favorecem a indicação de fragilidades estruturais como iluminação das vias públicas, calçadas em mau estado de conservação e limpeza insuficiente. Esses problemas apontam para uma negligência tanto no cuidado com o espaço público quanto na prevenção de acidentes. A manutenção e o planejamento urbano desempenham um papel essencial na segurança de todos, mas especialmente na proteção de grupos mais vulneráveis. Somado a isso, é necessário considerar condições como síncope, labirintopatias, problemas cardíacos, fatores intrínsecos estes, comuns em idosos.

A síncope é uma perda transitória da consciência provocada por uma redução temporária do fluxo sanguíneo cerebral e, portanto, um fator significativo para quedas em idosos. Esse evento pode contribuir para o medo e para a perda da perspectiva de autonomia e confiança em estar só, independentemente do local ou ambiente. As causas da síncope podem variar e incluem disfunções autonômicas, hipotensão ortostática, entre outras comuns em idosos^(23)^.

Já as labirintopatias são distúrbios que afetam o labirinto e podem provocar tontura, vertigem e desequilíbrio, frequentemente associados à dificuldade em realizar tarefas diárias que requerem coordenação motora. Em idosos, tendem a ser mais prevalentes devido ao envelhecimento natural do sistema vestibular e à maior incidência de doenças crônicas que podem comprometer sua função^(24)^.

Os problemas cardíacos também desempenham papel relevante na gênese das quedas. Condições como bradicardia podem reduzir o fluxo sanguíneo cerebral e levar a episódios de tontura e desmaio. O bloqueio atrioventricular (BAV) é outro fator que pode resultar em bradicardia significativa e consequente risco de quedas^(23)^.

O envelhecimento também está associado a fatores como sarcopenia, osteoporose e diminuição da capacidade sensorial, o que agrava os efeitos dessas condições. A presença de multimorbidades e a polifarmácia, frequentemente observadas em idosos, potencializam o risco de quedas^(25)^.

As condições ideais para ações, intervenções e prevenção de quedas em idosos incluem a identificação e o manejo adequado dos fatores intrínsecos e extrínsecos. Isso envolve avaliação clínica regular, tratamento das condições de base, acompanhamento das condições cardíacas e promoção de atividades que melhorem o equilíbrio, a força muscular e a flexibilidade. Importante ainda atentar para a instalação de iluminação adequada, pisos antiderrapantes, assentos com anteparos para maior segurança durante o banho, instalação de barras de apoio, organização cuidadosa dos ambientes e remoção de tapetes soltos^(26)^. Quedas em pessoas idosas estão frequentemente associadas a fatores como degraus, desníveis, tapetes, animais de estimação e objetos pelo chão^(27)^. Assim, por meio da adoção de práticas como a reorganização dos ambientes, orientações educativas e adequações no espaço físico, é possível minimizar os riscos de acidentes por causas externas, entre eles as quedas.

Entre os idosos pesquisados, a queda relacionou-se a uma busca por autonomia e utilidade. Emergiu a percepção social que associa o envelhecimento à fragilidade e à dependência, o que os incentivava a permanecerem ativos, mesmo em situações de risco. Desde a realização de uma tarefa doméstica simples, os idosos frequentemente desafiam seus próprios limites físicos, talvez em uma tentativa de resistir ao preconceito e de afirmarem a própria independência e autonomia.

Nesse contexto, são, portanto, necessárias orientações para prevenir esse evento nesta população, com ênfase nos fatores extrínsecos, como a adaptação do domicílio, para possibilitar a segurança do ambiente^(28)^. Imperativo também orientar os idosos e suas famílias acerca dos riscos e consequências, por meio da promoção da saúde, a fim de corrigir ou minimizar os fatores de risco.

### Limitações do estudo

Embora o estudo tenha potencial de ser replicado e ampliado em outras ESFs, foram pesquisados apenas idosos de uma Estratégia de Saúde da Família.

### Contribuições para a área da enfermagem, saúde ou política pública

Conhecer a relação entre o evento queda de idosos da comunidade e suas percepções de qualidade de vida possibilita a adoção de medidas preventivas, tanto individuais quanto coletivas, o que, possivelmente, impactará nas situações de fragilidade e vulnerabilidade desses idosos e reduzirá internações hospitalares e desfechos desfavoráveis, como sequelas irreparáveis e óbito. Analisar os fatores, conforme a realidade dos idosos que vivem em comunidade, pode favorecer uma atenção integral, abrangente e mais resolutiva em todos os níveis de prevenção. Essas estratégias reforçam a implantação, implementação e manutenção da autonomia dos idosos e possibilitam o convívio familial e social, o que suscita reflexões acerca de práticas voltadas à prevenção.

## CONSIDERAÇÕES FINAIS

A relação entre quedas em idosos e as percepções de qualidade de vida é complexa, uma vez que esse evento impacta diretamente na saúde física e emocional, bem como acentua a fragilidade e a vulnerabilidade desse grupo populacional.

Muitos idosos tendem a naturalizar essa ocorrência, influenciados por perspectivas sociais que associam o envelhecimento à inevitabilidade da vulnerabilidade e fragilidade. Essa visão reforça a necessidade de estratégias que promovam a sensibilização acerca dos riscos e consequências das quedas, ao mesmo tempo em que incentivem práticas de prevenção e cuidado. A valorização de redes de apoio social e espiritualidade mostrou-se relevante para o enfrentamento das repercussões das quedas, sendo um recurso essencial na busca por melhores percepções de qualidade de vida.

Nesse contexto, sugere-se aos profissionais de saúde a produção do conhecimento direcionado aos idosos, por meio de pesquisas de intervenções em prol de prevenir este evento, que possibilitem gerenciar a fragilidade e vulnerabilidade.

## Data Availability

Os dados da pesquisa estão disponíveis no corpo do artigo.
